# Tuberculosis infection versus anti-tumor necrosis factor therapy: screening challenges in psoriatic patients

**DOI:** 10.3109/21556660.2012.744315

**Published:** 2012-10-26

**Authors:** Caius Solovan, Elena Chiticariu, Adelina Timofte, Irina Stoia-Djeska

**Affiliations:** 1 Department of Dermatology, University of Medicine and Pharmacy “Victor Babeş”, TimisoaraRomania; 2 Department of Dermatology, County Hospital, TimişoaraRomania; 3 Department of Pulmonary Medicine, Victor Babeş Hospital, TimişoaraRomania

**Keywords:** Interferon gamma, Psoriasis, Tuberculin skin test, Tuberculosis infection, Tumor necrosis factor

## Abstract

**Objectives:**

The aim of this study was to analyze the performance of the tuberculin skin test (TST) for screening and monitoring patients treated with anti-tumor necrosis factor agents, in a high-incidence area.

**Methods:**

A 3-year retrospective study was carried out on 268 subjects. The study included 68 patients with moderate-to-severe psoriasis, screened for latent tuberculosis infection (LTBI) and subjects without psoriasis (100 adults and 100 children) with close contact with infected individuals.

**Results:**

Positive tuberculin skin test (TST) results (induration >5 mm) were observed in 70.5% (48/68) of patients with psoriasis, higher than those observed in subjects with suspicion of tuberculosis or with close contact with infected individuals: 51% (51/100) in the adult group and 30% (30/100) in the children group.

**Conclusions:**

These results show that the prevalence of LTBI evaluated with the TST in the psoriatic group is higher than in subjects without psoriasis.

**Limitation:**

The positive reactions were not confirmed by other verification methods.

## Introduction

The introduction of biologic therapy has increased the incidence of tuberculosis (TB) infections, all tumor necrosis factor (TNF) antagonists being associated with activation of latent tuberculosis infection^1^. The need for latent tuberculosis infection (LTBI) screening has raised many questions about which is the most precise test. The tuberculin skin test (TST) is still widely used, but it suffers from several limitations, of which its low specificity is the most important. Although the TST is still considered a useful method, it is difficult to carry out and read in psoriatic patients who had extensive lesions, because these patients barely present clinically unaffected skin^2^. However, we believe that not only the affected skin influences the TST results, but also even the apparently healthy skin of psoriatic patients can be the site of immunological processes. Tuberculin hyperergy seems to be common in psoriatic patients. In this regard we analyzed the performance of the TST for screening patients treated with anti-tumor necrosis factor agents, in a high-incidence area.

## Subjects and methods

A 3-year retrospective study was carried out between 2009 and 2011. We compared 68 adult psoriatic patients who had moderate-to-severe psoriasis with two groups of subjects without psoriasis, who had been in close contact with active tuberculosis patients: 100 adults and 100 children.

Moderate-to-severe psoriasis was defined as a Psoriasis Area and Severity Index (PASI) or a Body Surface Area (BSA) >10. Subjects older than 31 years were selected and classified as adults, subjects younger than 15 years were classified as children. Close contact was defined as a close interaction with an active tuberculosis patient. The diagnosis and evaluation of psoriasis were performed by experienced dermatologists from our Dermatology Clinic. The two groups of individuals without psoriasis were evaluated by experienced pneumologists from the Pneumology Clinic.

LTBI screening was performed by pneumologists according to: epidemiology, history of Bacillus Calmette-Guérin (BCG) vaccination, chest X-ray, and tuberculin skin test (TST) with 2 units of purified protein derivate (PPD). The cut-off for a positive skin test was accepted as an area of induration of more than 5 mm.

For statistical analysis we used InStat software from GraphPad (InStat version 3.06, GraphPad Software Inc, San Diego, CA, USA). A descriptive analysis was performed for the whole sample. A *p*-value of <0.05 was considered statistically significant.

The study was conducted in accordance with institutional review board requirements and the Declaration of Helsinki ethical principles.

## Results

We reviewed the medical records of 68 psoriatic patients (mean age: 50 years, SD 11.9; male/female ratio 1.3:1) admitted to the Dermatology Clinic between 2009 and 2011. The results were compared with those of 200 non-psoriatic subjects from the Pneumology Clinic: 100 adults (mean age: 50.3 years, SD 11.8; male/female ratio 1:1.2) and 100 children (mean age: 12.7 years, SD 1.6; male/female ratio 1:1.2).

Positive TST results were observed in 70.5% (48/68) of patients with psoriasis, higher than those observed in the control groups: 51% (51/100) in non-psoriatic adults (*p* < 0.05) and 30% (30/100) in non-psoriatic children (*p* < 0.001). The mean value of TST was as follows: 9.6 mm in psoriatic patients, 6.4 mm in non-psoriatic adults group, and 4 mm in non-psoriatic children (*p* < 0.01).

## Discussion

Our results show that the prevalence of LTBI evaluated with the TST in the psoriatic group is higher than in subjects without psoriasis who had a higher risk of tuberculosis infection.

The risk of tuberculosis raises many questions about the safety of anti-TNF agents. Two tests are currently used for detecting LTBI in our country: the TST and QFT, the latter an IFN-gamma release assay (IGRA). Each of these tests relies on a different immune response: the TST evaluates *in vivo* delayed-type hypersensitivity, and QFT assesses *in vitro* release of interferon gamma (IFN–γ)^3^. Because of the high cost of QFT, this is not a routine test in many countries, the TST remaining the standard screening method.

The TST is accompanied by false-positive and false-negative results. The causes of false-positive reactions may include prior BCG vaccination and infections with non-tuberculosis mycobacteria. False-negative results are also possible in the presence of chronic inflammation or immunosuppression^4^. In addition to these disadvantages, the test is less accurate in patients with psoriasis^5^. We have chosen patients older than 31 years of age because the BCG vaccination is given routinely to children in our country, an endemic area. According to our national immunization calendar, BCG vaccine is given at birth and at age 14 years. BCG-mediated protective immunity lasts only for 10–15 years^6^, after which these subjects are no longer under the vaccine protection. Positive TST results were more common in patients with psoriasis compared with non-psoriatic children (more likely to be under BCG vaccine protection), which means that positive results due to BCG vaccination are low.

The guidelines recommend the use of interferon-gamma release assays (IGRAs) for screening – tests developed as an alternative method to the TST. These assays detect either the frequency of activated specific T-cells releasing IFN–γ isolated from peripheral mononuclear cells, or the amount of IFN–γ released in response to *M. tuberculosis* proteins. These antigens are encoded by mycobacterium-specific genes, which are not shared with the BCG vaccine strains or non-tuberculosis mycobacterium and are believed to be more specific than the TST^7^. Because of these characteristics, false-positive reactions due to BCG vaccination and atypical mycobacterium are limited, but IFN–γ is known to be a crucial mediator in the cytokine cascade of psoriasis. Considerable amounts of IFN–γ-positive cells were found in psoriatic skin; also the expression of interleukin–12 is increased in psoriatic lesions^8^. Psoriasis was also associated with elevated serum levels of IFN–γ^9^, which raises questions about the specificity of IGRAs in psoriatic patients.

Another factor that may overestimate the positive TST results is the Koebner phenomenon. This reaction represents the development of psoriatic lesions in the traumatized non-lesional skin^10^. The lesions triggered by the injection injury could be interpreted as positive reactions. Dogan *et al*. reported the development of the Koebner phenomenon after intradermal injection of PPD in psoriatic patients. The authors also observed that the antigen was more effective in inducing the Koebner phenomenon than injury alone^11^.

The study has some limitations. It was retrospective and included a relatively low number of patients with psoriasis. The positive reactions were not confirmed by other verification methods, such as the use of IGRAs. Thus, we could not identify false-positive results by correlation with other tests. We excluded false-positive TST results caused by BCG vaccination, the test being performed at least 17 years after vaccination.

The results raise questions about the most accurate approach in endemic areas. There are two possibilities: either each patient with psoriasis must receive chemoprophylaxis before starting anti-TNF therapy, or other tests should be used. The first scenario is far from being ideal. The most significant inconveniences are the undesirable side-effects, cost, and the requirement for daily medication for 9 months^12^. The second possibility seems to be reasonable, but the economic implications limit the utilization of QFT as a screening test. In such a case, the cost–effectiveness ratio must be carefully evaluated. However, carefully monitoring of patients receiving biologic therapies still remains the best approach.

False-positive and negative results occur with both TST and IGRAs because both are indirect tests and the immune system might influence the results. Although published evidence suggests that IGRAs maintain their diagnostic specificity and sensitivity for LTBI better than TST, no test is perfect and these techniques are certainly no exception.

## Conclusion

Our study revealed that TST clearly overestimates the incidence of LTBI in psoriatic patients. Although TB incidence is still high in many countries, confirmatory tests are required to avoid unnecessary chemoprophylaxis. The results cannot be extrapolated to non-endemic areas, owing to the large variations in economic and epidemiological factors. Finally, efforts to improve LTBI detection in psoriatic patients need to be considered in both endemic and non-endemic countries.

## Transparency

### Declaration of financial/other relationships

There is no conflict of interest of any of the authors with the results of this study.
